# Influence of Partial Replacements of NaCl by KCl on Quality Characteristics and the Heterocyclic Aromatic Amine Contents of Bacon

**DOI:** 10.3390/foods11020143

**Published:** 2022-01-06

**Authors:** Hongzhen Du, Xiangao Li, Qiang Wang, Qian Liu, Qian Chen, Baohua Kong

**Affiliations:** College of Food Science, Northeast Agricultural University, Harbin 150030, China; dhz0531@163.com (H.D.); lixiangao67@163.com (X.L.); wangqiangneau@163.com (Q.W.); liuqian@neau.edu.cn (Q.L.); chenqianego7@126.com (Q.C.)

**Keywords:** bacon, heterocyclic aromatic amine, sodium replacements, oxidation, sensory quality

## Abstract

The influence of partial replacements of NaCl by KCl (0, 10, 20, and 30%) on the heterocyclic aromatic amine (HAAs) contents and quality characteristics of bacon were investigated. The Na^+^ content, moisture, a_w_, pH, L* value, and sensory saltiness decreased and K^+^ content, a* value, and sensory bitterness increased significantly with increased substituting rates of NaCl by KCl (*p* < 0.05). There were no significant differences between the control and KCl substitution samples for the b* value, redness, and sensory off-odor (*p* > 0.05). The creatine content was not affected by the different KCl-substituting rates during the marinating process (*p* > 0.05), but it diminished in the smoking and frying processes (*p* < 0.05). The increase in the KCl-substituting rates increased the total heterocyclic aromatic amine (HAA) contents in fried bacon (*p* < 0.05). Moreover, the nonpolar HAA content in bacon was higher than the polar HAA content (*p* < 0.05). In summary, the partial replacement of NaCl by KCl increased the total HAA content and led to changes in bacon quality.

## 1. Introduction

Sodium salt has been used as a preservative in meat products to reduce water activity, limit oxygen solubility, obstruct microbial growth, enhance flavor, and improve quality and texture [1,2]. A diet of high dietary sodium salt intake can promote the development of hypertension and cardiovascular disease [3]. Raw meat itself is low in sodium; however, meat products usually have a high sodium content. It is reported that approximately 20% of the daily sodium intake comes from meat products [4]. Among meat products, cured meat and sausages have the highest salt contents [5]. There is currently an increasing demand to reduce the salt contents of meat products. Generally, directly reducing the sodium chloride (NaCl) level is the most convenient method; however, sodium reduction may decrease the sensory qualities, shorten the shelf life, and increase the thiobarbituric acid reactive substances (TBARS) and carbonyl contents of meat products [6]. Another common strategy for reducing the NaCl content is partial replacement by other chloride salts, such as potassium chloride (KCl). Numerous studies have been conducted on KCl replacing NaCl in meat products [2,7]. Li et al. [8] reported that the partial replacement of NaCl by KCl did not significantly impact the amount of polycyclic aromatic hydrocarbons (PAHs) in bacon.

Heterocyclic aromatic amines (HAAs) are heat-induced carcinogens. Nagao et al. [9] first found HAAs from charred fish and meat. To date, more than 30 HAAs have been isolated and identified in food [10] and at least 25 HAAs identified as potent mutagens [11]. According to the heating temperature, HAAs are subdivided into thermic HAAs (100–300 °C) and pyrolytic HAAs (>300 °C) [12]. Another classification of HAAs subdivides them into IQ-type HAAs and non-IQ-type HAAs, according to their chemical structures. IQ-type HAAs are produced from the reaction of free amino acids, creatine/creatinine, and hexoses during heating, and non-IQ-type HAAs are generated from the pyrolytic reaction between amino acids and proteins [12]. In addition, carnosine or anserine may act as precursors of 3-amino-1,4-dimethyl-5H-pyrido[4,3-b]indole (Trp-P1), 3-amino-1-methyl-5H-pyrido[4,3-b]indole (Trp-P2), 2-amino-9H-pyrido[2,3-b]indol (AαC), and 2-amino-3-methyl-9H-pyrido[2,3-b]indol (MeAαC) [11]. Generally, temperature, heat transfer, and heating conditions have a significant impact on the formation of HAAs [13]. Other factors that affect HAA formation are muscle types [14], pH [15], sugar [16], amino acids [17], fat contents and lipid oxidation [13], and antioxidants and reducing agents [18,19]. However, there are few reports on the influence of NaCl substitutes in brine on the formation of HAAs in meat products.

Bacon is a highly consumed meat product known for its distinctive texture, flavor, and particular sensory characteristics [20]. It is processed from pork bellies. The skinned bellies are injected with brine, then smoked using woodchips [21]. The purpose of this study was to evaluate the influence of a partial substitution of NaCl by KCl on the HAA contents and quality characteristics of bacon. The contents of HAAs in smoked bacon were analyzed by ultra-high-performance liquid chromatography-triple quadrupole mass spectrometry (UHPLC-MS/MS). The moisture content, water activity, color, TBARS, and carbonyl contents were measured to evaluate the effects of the quality characteristics in bacon after a partial substitution of NaCl by KCl.

## 2. Materials and Methods

### 2.1. Materials

The HAA standards, including 2-amino-1-methyl-6-phenylimidazo[4,5-b]-pyridine (PhIP), 2-amino-3-methyl-imidazo[4,5-f]-quinoline (IQ), 2-amino-3-methyl-imidazo[4,5-f]-quinoxaline (IQx), 2-amino-3,4-dimethyl-imidazo[4,5-f]-quinoline (MeIQ), 2-amino-3,4,8-trimethyl-imidazo[4,5-f]-quinoxaline (4,8-DiMeIQx), 2-amino-3,7,8-trimethyl-Imidazo[4,5-f]-quinoxaline(7,8-DiMeIQx), AαC, MeAαC, 1-methyl-9H-pyrido[3,4-b]indole(Harman), 9H-pyrido[3,4-b]indole(Norharman), 2-amino-3,8-dimethyl-3H imidazo [4,5,f] quinoxaline (MeIQx), and 2-amino-5-phenylpyridine(Phe-P-I). 2-amino-3,4,7,8-tetramethylimidazo[4,5-f]-quinoxaline (TriMeIQx), were applied as the internal standard. These standards were obtained from Toronto Research Chemicals (Downsview, ON, Canada) and were of HPLC grade (purity > 98%). Solid-phase extraction cartridges (Oasis MCX cartridges, 3 cm^3^/60 mg) were purchased from Waters (Shanghai, China). Ethanol, methanol, and acetonitrile (HPLC grade) were purchased from Fisher Scientific (Shanghai, China). Ammonium acetate, dichloromethane, and n-hexane (HPLC grade) were purchased from Sigma-Aldrich (Shanghai, China). Trichloroacetic acid, disodium hydrogen phosphate, sodium dihydrogen phosphate, hydrochloric acid, acetone, ethyl acetate, ethanol, chloroform, and 2-thiobarbituric acid (TBA) were analytical grade (AR) and purchased from Sinopharm Chemical Reagent Co., Ltd. (Shenyang, China).

### 2.2. Bacon Preparation

Landrace × Large white × Duroc crossbred pigs (90–100-kg body weight, 6 months old) were harvested at a local processing plant. Animals were humanely slaughtered in a commercial abattoir (Beidahuang Meat Corp., Harbin, Heilongjiang, China) following the standard industry procedures. Sixty pork bellies were obtained from pig carcasses within 24 h after slaughter and divided into three groups of 20 belly chops each. Each group corresponded to an independent process. The average length, width, and thickness of each belly chop were 25, 15, and 2.5 cm, respectively. The bacon was processed according to the method of Du et al. [20], with some modifications. There were four NaCl substitution (SS) levels (treatments), including 100% NaCl (control, CS), 90% NaCl + 10% KCl (SS1), 80% NaCl + 20% KCl (SS2), and 70% NaCl + 30% KCl (SS3). Each group (20 belly chops) was a batch. There were four treatments in each batch, and each treatment had five belly chops. The brines contained 500-mg/L sodium nitrite and 90-g/L salts (NaCl + KCl). The above four salt concentrations of brine (CS, SS1, SS2, and SS3) were injected (approximately 10% of the meat weight) into each treatment group by an injector (Jiaxing Expro Industrial Co., Ltd., Zhejiang, China), and then, the bellies were immersed in the above four brines at 4 °C for 20 h. The bellies were first dried in a smokehouse (Jiaxing Expro Industrial Co., Ltd., Zhejiang, China) at 45 °C for 30 min and then smoked with oak woodchips at 55 °C and 65 °C for 1 h and 2 h, respectively. Subsequently, the bacon was removed from the smokehouse and cooled for 4 h at 4 °C and then frozen at −20 °C. The frozen bacon was sliced into 2 mm thick slices by a slicer (Jiaxing Expro Industrial Co., Ltd., Jiaxing, China) and vacuum-packaged. Each package contained eight slices of bacon (150 ± 6 g). Six vacuum packages were obtained for each belly chop, and five belly chops for each treatment were obtained for a total of 30 packages of bacon. Finally, the packaged bacon was stored at −18 °C and tested within 7 days.

### 2.3. Measurement of K^+^ and Na^+^ Contents, Moisture Content, Water Activity, and pH

The Na+ and K+ contents were measured using the method of Zhang et al. [22]. The moisture content and water activity (a_w_) of the samples were detected according to the method described by Sun et al. [23]. The pH was measured using a pH meter (Mettler Toledo Instruments Co., Ltd., Shanghai, China) [24].

### 2.4. Color Measurement

The lean parts of the sliced bacon were used to measure color with a ZE-6000 colorimeter (Juki Corp., Tokyo, Japan) using a D65 light source and a 10° observer with an 8-mm diameter measuring area and a 50-mm diameter illumination area according to the method described by Zhang et al. [24].

### 2.5. Measurement of Thiobarbituric Acid Reactive Substance and Carbonyl Content

The fat parts of the sliced bacon were used for a thiobarbituric acid-reactive substance (TBARS) analysis based on the method of Zhang et al. [25]. The TBARS value was expressed as mg of malonaldehyde (MDA)/kg of sample. The lean parts of the sliced bacon were used to determine the carbonyl content according to the description by Zhang et al. [25]. The carbonyl content was expressed as nmol/mg protein and calculated by an absorption coefficient of 22,000 M^−1^·cm^−1^.

### 2.6. Sensory Evaluation

The assessors were recruited from faculty, staff, and graduate students (Northeast Agricultural University). The assessment was performed in three sessions. There were 12 panelists in total, and they assessed across the sessions. Twelve packages of sliced bacon were used in each panelist test. Before the sensory evaluation, the recruited assessors were trained for approximately 3 h in each session. The attributes (saltiness, redness, bitterness, and off-odor) were selected based on previous studies [26,27,28]. A seven-point scale was employed with 7 representing “extremely intense”, 4 representing “average intense”, and 1 referring to “none” for all attributes [20]. The salt solutions consisting of 2.5 g/L and 25 g/L salt and hydrochloride solutions consisting of 0.001 g/L and 0.01 g/L quinine hydrochloride were prepared for saltiness and bitterness training, respectively. A light pink to dark red card was prepared for redness training. The varying levels of oxidized corn oil were used to coat and pan-fry the bacon slices for off-odor training. The slices were pan-fried using a saucepan for about 2 min at 150–170 °C. Subsequently, the fried bacon was cut into 1 cm × 3 cm pieces and kept in an oven at 45 °C to maintain their temperature.

### 2.7. Measurement of Creatine Content

The creatine content in the samples was measured using the method of Zhang et al. [29]. The creatine content was expressed as mg/g dry matter of the samples.

### 2.8. Determination of HAAs

The samples were pan-fried for 2 min at 150–170 °C in a saucepan before analysis. The contents of the HAAs were determined according to the methods used by Zhang et al. [25]. The samples (2.0 g) were chopped and placed in a 50-mL centrifuge tube. The internal standard (TriMeIQx) working solution (0.2 mL, 200 μg/L) and 9.8-mL solution of sodium hydroxide (NaOH) (1 M) and methanol (7:3, *v*/*v*) were added, and the samples were subsequently homogenized for 1 min. The head of the homogenizer was washed with a solution of 10 mL of 1-M NaOH and methanol (7:3, *v*/*v*), and the washing solution was merged into the sample extraction centrifugal tube. The extracts were centrifuged for 10 min at 10,750× *g*, and the supernatant was collected. The Waters Oasis MCX cartridges (3 cm^3^/60 mg) were previously preconditioned with 2 mL methanol and 3 mL of 0.1 M NaOH and then loaded with 5 mL of the extract solution. The cartridges were washed with a mixture of 0.1-M NaOH and methanol (45:55 *v*/*v*) and 2-mL n-hexane. The HAAs absorbed in the cartridge were finally eluted by 1.5 mL of methanol and dichloromethane (1/9, *v*/*v*). Finally, the eluate was concentrated to dryness by nitrogen at 30 °C and dissolved with a solution of 1.0 mL acetic acid buffer and acetonitrile (1:1, *v*/*v*), then filtered through a 0.22 μm microporous filtration membrane (Anpel, Shanghai, China) before analysis.

The concentrations of the HAAs in the bacon samples were determined using the ultra-high-performance liquid chromatography tandem triple quadrupole mass spectrometer LCMS-8050 system (UHPLC-MS/MS) (Shimadzu, Shenyang, China) equipped with a C18 Shim-pack XR-ODS III (2.2 μm, 2.0 mm × 150 mm). The mobile phase was 10-mM ammonium acetate (A) and acetonitrile (B) at a flow rate of 3 mL/min. The gradient elution program was: 0–3.5 min, 60% B, 40% A; 3.5–5 min, 60–90% B, 10–40%; 5–8 min, 90% B, 10% A; 8–8.1 min, 90–10% B, 10–90% A; and 8.1–13 min, 10% B, 90% A. The standard solution contained HAAs at 0.1, 0.5, 1, 5, 10, 20, and 50 μg/L. The internal standard was 4,7,8-TriMeIQx (20 μg/L), and the injection volume was 2 μL.

### 2.9. Statistical Analysis

The effects of different concentrations of NaCl on HAA contents and quality characteristics of bacon were evaluated by SPSS statistics 22.0 (SPSS, Chicago, IL, USA). Analysis of Variance (ANOVA) was performed with Duncan’s multiple comparison to assess the significance level with a threshold of *p* < 0.05. The results were expressed as the mean ± standard error (SE). There are three groups of belly chops, and each group (20 belly chops) was a batch. Three independent batches of smoked bacon (replicates) were used. The HAA data and physicochemical properties of bacon were analyzed using a mixed model. Each replicate (*n* = 3) was taken as a random effect in this model, and the different treatments were taken as fixed effects. The same panelists were used in all the sessions. The sensory evaluation data were also analyzed using a mixed model where the different treatments, sensory attributes, and panelist numbers (*n* = 12) were taken as the fixed effects, and the session number (*n* = 3) was taken as the random effect.

## 3. Results and Discussion

### 3.1. K^+^ Content, Na^+^ Content, Moisture Content, Water Activity, and pH

The results of the K^+^ content, Na^+^ content, moisture content, a_w_, and pH value of bacon are shown in Table 1. The K^+^ and Na^+^ contents of the control samples, SS1, SS2, and SS3 were 0.08, 0.17, 0.29, and 0.40 g/100 g and 1.05, 0.89, 0.75, and 0.62 g/100 g, respectively. The K^+^ content of bacon treated with SS3 was significantly higher than those of the other three treatments (*p* < 0.05), while the Na^+^ content of bacon treated with SS3 was significantly lower than those of the other three treatments (*p* < 0.05). This certainly occurred as the KCl contents in brine increased and NaCl contents decreased. In addition, with the increased KCl substitution rate in brine, the total content of K^+^ and Na^+^ in bacon decreased. This may be due to the fact that the same mass of KCl has a smaller molar concentration than that of NaCl, thus reducing the total molar concentration in the brine. The lower molar concentration in brine had a lower osmotic pressure in the salt solution, which led to a slower transfer of salt ions from the brine into the muscle cell [30]. Zhang et al. [22] also found that the total molar concentration of KCl and NaCl in salted pork declined with an increased KCl substitution rate in brine.

The moisture content of the control samples was significantly higher than those of the other three treatments (*p* < 0.05), and there were no significant differences between the SS1, SS2, and SS3 samples. Generally, a higher salt concentration will cause water loss from the muscle. However, K^+^ ions have a negative hydration effect, which destabilizes protein conformation and increases water loss [22]. In addition, Zhang et al. [22] also reported that the gap between the muscle fibers of the muscle bundle increased when the replacement rate of KCl increased. Therefore, the free water, which exists in the space between fiber bundles, was easier to evaporate during smoking. The a_w_ and pH of the control samples were higher than those of bacon treated with the other three groups (*p* < 0.05). With an increased KCl-substituting rate in brine, there was no significant difference in a_w_ between the SS1, SS2, and SS3 treatment samples (*p* > 0.05), while the pH of bacon treated with SS3 was lower than that of bacon treated with SS1 and SS2 (*p* < 0.05). The decrease in the a_w_ of bacon treated with KCl substitution was due to the decreased moisture content.

### 3.2. Color

External quality criteria such as color is an important indicator reflecting the physiological, biochemical, and microbial changes of muscles [31]. The changes in the color of bacon treated with different KCl substitution rates in brine are shown in Table 1. The control samples had the highest L* value compared with the other three treatment groups (*p* < 0.05), and there were no significant differences in the L* value between the SS1, SS2, and SS3 samples. The decrease in the L* value of the SS1, SS2, and SS3 samples may be because of the decline in the moisture content in these samples. The a* value of the control samples and SS1 was significantly lower than those of bacon treated with SS2 and SS3 (*p* < 0.05), and there were no significant differences between the SS2 and SS3 samples (*p* > 0.05). This result revealed that the partial replacement of NaCl by KCl could increase the a* value of the sample after smoking. Muoz et al. [32] also found that the a* value of the smoked salmon (*Salmo salar*) with a 25% KCl replacement of NaCl was significantly higher than that of the control. There was no significant difference in the b* value for bacon treated with different KCl substitutions in brine (*p* > 0.05). dos Santos et al. [33] found that the b* value of fermented cooked sausages cured with a 50% KCl and 75% KCl replacement of NaCl was not significantly different to the control group. Li et al. [8] also found that the b* value of bacon cured with a 25% KCl replacement of NaCl was not significantly different from the control group.

### 3.3. TBARS and Carbonyl Content

**Figure 1:**
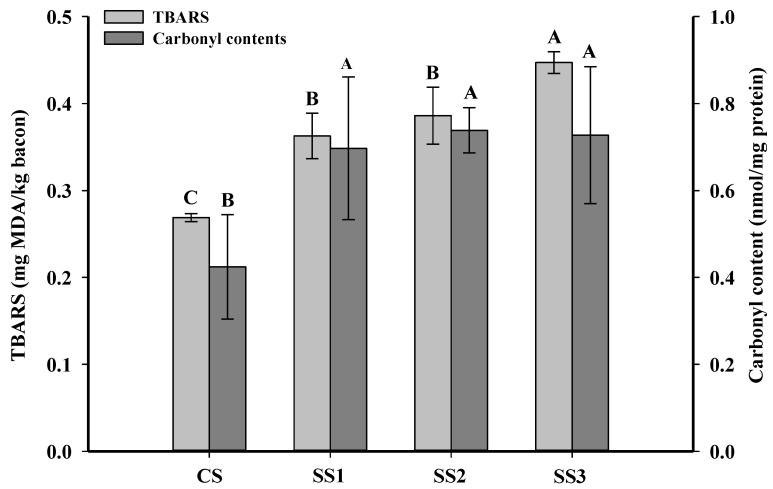
Influence of the partial replacement of NaCl by KCl on the TBARS and carbonyl contents of bacon. ^A–C^ Means in the TBARS or carbonyl contents within different KCl-substituting rates are significantly different (*p* < 0.05). CS, control (100% NaCl); SS1, 90% NaCl + 10% KCl; SS2, 80% NaCl + 20% KCl; and SS3, 70% NaCl + 30% KCl. TBARS: thiobarbituric acid reactive substance.

Oxidative rancidity is a major quality problem of bacon during chilled storage. The TBARS value can indicate the level of the second product of lipid peroxidation in meat and meat products [34]. Yildiz [35] showed that meat and meat products with less than 3-mg MDA/kg meat were defined as acceptable products. As shown in Figure 1, the TBARS values of the control samples, SS1, SS2, and SS3 were 0.27-, 0.37-, 0.39-, and 0.45-mg MDA/kg meat. The TBARS value of the control samples was significantly lower than that of the other three treatments (*p* < 0.05), and there were no significant differences between the samples treated with SS1 and SS2 (*p* > 0.05). The lower TBARS value in the control samples may be due to the decreased solubility of oxygen in the high ionic strength solutions [36]. Ripollés et al. [7] also found that 100% NaCl hams had the lowest TBARS values in comparison with a 50% KCl replacement of NaCl.

The content of carbonyl compounds in meat and meat products is the primary indicator of protein oxidation [37]. The changes in the carbonyl contents of bacon treated with different KCl substitution rates in brine are shown in Figure 1. The carbonyl contents of the control bacon were significantly lower than those of the other three treatments (*p* < 0.05), and there were no significant differences between the samples treated with SS1, SS2, and SS3 (*p* > 0.05). The increase in the carbonyl contents of the SS1, SS2, and SS3 samples was possibly because the K^+^ ions can enhance the surface tension of proteins and facilitate more slack protein structures [22], further accelerating the oxidation of muscle proteins. However, previous studies on dry sausage and dry-cured bacon have shown that the partial replacement of NaCl with KCl could significantly diminish the carbonyl contents [1,38]. These differences are perhaps attributed to the high temperatures used during smoking. Gan et al. [39] found that the carbonyl content of bacon with a 30% KCl replacement of NaCl was not different from that of the control after marination, while its carbonyl content was significantly higher than that of the control after roasting and ripening.

### 3.4. Sensory Evaluation

The data of the mean scores and standard error for the attributes (saltiness, redness, bitterness, and off-odor) are shown in Table 2. The saltiness of the bacon treated with SS3 was significantly lower than that of the other three treatments (*p* < 0.05), and there were no significant differences between the samples treated with CS, SS1, and SS2 (*p* > 0.05). The lower scores for saltiness in the SS3 treatment may be related to the increased KCl substitution rates, which reduced the total molar concentration in bacon (Table 1). The SS3 treatment had a higher score for bitterness than that of the other three treatments (*p* < 0.05), and there were no significant differences between the samples treated with CS, SS1, and SS2 (*p* > 0.05). The higher scores for bitterness in the SS3 treatment may be related to the KCl substitution. Petracci et al. [40] reported that over a 30% KCl substitution rate will give a bitter and metallic taste to products. The sensory evaluation revealed that there were no significant differences in the off-odor and redness between the different treatments. Chen et al. [41] also found that the color of dry sausage was not affected by NaCl substitution with KCl.

### 3.5. Creatine Content

**Figure 2:**
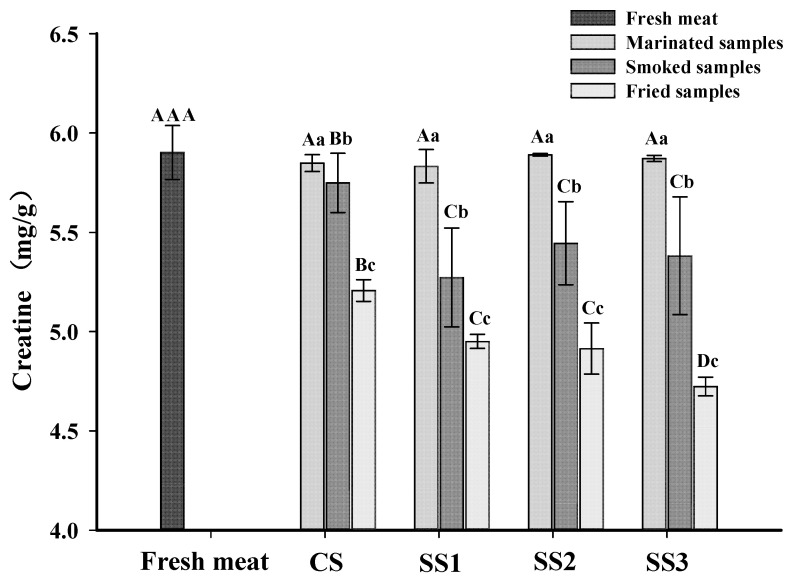
Influence of the partial replacements of NaCl by KCl on the creatine contents of bacon at different processing stages. ^A–D^ The differences between the creatine content in bacon at the same processing stage with different KCl-substituting rates and the content of creatine in fresh meat (*p* < 0.05). ^a–c^ The differences of the creatine content in bacon at the same processing stage (*p* < 0.05). CS, control (100% NaCl); SS1, 90% NaCl + 10% KCl; SS2, 80% NaCl + 20% KCl; and SS3, 70% NaCl + 30% KCl.

Creatine is a non-protein nitrogen compound that exists in the form of phosphocreatine in the muscle of vertebrates and is an essential precursor of IQ-type HAAs [29,42]. Heating transforms creatine into the cyclization product creatinine, which further forms an imidazole ring [13]. The creatine contents of each sample for the different processing stages are shown in Figure 2. The content of creatine in the marinated samples was not different from fresh meat (*p* > 0.05), and there were no significant differences in the creatine contents between bacon cured with different substitution rates of KCl (*p* > 0.05). This result revealed that the creatine content in the marinated samples was not affected by the KCl and NaCl concentrations in the brine. The content of creatine in fresh meat was higher than in smoked and fried bacon (*p* < 0.05), and the content of creatine in fried bacon was lower than that in smoked bacon (*p* < 0.05). The decline of the creatine content in smoked and fried bacon is likely due to the conversion of creatine to creatinine during processing [43]. Gibis et al. [44] also found that the creatine content in fried bacon decreased with the increased frying times at higher heating temperatures. However, the creatine content of the smoked samples treated with CS was higher than those of the other three samples, and there were no significant differences in the creatine content between bacon cured with different substitution rates of KCl in brine (*p* > 0.05). In addition, the fried samples treated with SS3 had the lowest creatine content compared to the other samples (*p* < 0.05), and there were no significant differences among the fried bacon treated with SS1 and SS2 (*p* > 0.05). These results revealed that the degradation reaction of creatine is perhaps related to the content of KCl in bacon. With the increased KCl substitution rate in the brine, the degree of muscle protein oxidation was increased (Figure 1). Moreover, the gap between the muscle fibers of the muscle bundle increased when the KCl replacement rate was large [22]. Therefore, creatine is released more easily from muscle to participate in the formation of creatinine.

### 3.6. HAAs Contents

Table 3 shows the effects of different substitution rates of KCl on the HAAs content of pan-fried bacon. Nine types of HAAs were identified in the pan-fried bacon. Three HAAs (MeAαC, MeIQ, and Phe-P-I) were not detected in all the samples. Dong et al. [45] also did not find MeAαC and MeIQ in commercially available sausage and bacon. The total HAA contents in pan-fried bacon ranged from 11.87 to 25.96 ng/g, which were comparable with the results of Gibis et al. [44]. Lu et al. [46] found that the roasted bacon had similar contents to the total HAAs with our study, while the crispy bacon had a lower total HAAs content. The present results were also higher than those reported by Soladoye et al. [47], who found that the total HAA content was 5.99 ng/g in fried bacon. Furthermore, the total HAA contents of bacon in the present study were much lower than those in the sausages and poultry products reported by Yang et al. [48] and Zhang et al. [29], respectively. These differences were presumably due to the different processing conditions (smoking, roasting, and frying); meat species; the thickness of the bacon slices; and heating temperature [12,44]. On the other hand, the content of the total HAAs in the CS samples (11.87 ng/g) was significantly lower than those of the other three samples (*p* < 0.05), and there were no significant differences between the samples treated with SS1 and SS2 (*p* > 0.05). The SS3 samples had the highest total HAAs content (*p* < 0.05). These results indicated that the increase of the KCl content in bacon can promote the formation of HAAs. This may be because some of the precursors in bacon, such as creatine (Figure 2), are released more easily from muscle when the KCl replacement rate increases.

As shown in Table 3, the nonpolar HAA (Norharman, Harman, and AαC) contents were higher than the polar HAA (IQ, IQx, 4,8-DiMeIQx, 7,8-DiMeIQx, MeIQx, and PhIP) contents in all the samples (*p* < 0.05). This indicated that the primary accumulation in the pan-fried processing was of nonpolar HAAs. Gibis et al. [44] and Yin et al. [49] found similar results in pan-fried bacon and smoked sausages. In addition, the contents of the nonpolar HAAs in the CS samples were significantly lower than those of the other three treatments (*p* < 0.05), and there were no significant differences between the SS2 and SS3 samples (*p* > 0.05). Moreover, the contents of the polar HAAs in bacon treated with SS3 were significantly higher than those of the other three samples (*p* < 0.05). Overall, the KCl replacement of NaCl in the brine had a significant influence on the contents of the nonpolar HAAs in bacon, which, in turn, affected the total HAA contents.

Among all of the HAAs, Norharman was the most abundant HAA in each treatment, consistent with the reports by Wang et al. [50] and Khan et al. [18]. The Norharman content in the CS samples was significantly lower than those of the other three samples (*p* < 0.05), and there were no significant differences between the SS2 and SS3 samples (*p* > 0.05). This may be due to the fact that the presence of KCl in bacon increases the contents of carbonyl compounds and aldehyde compounds (Figure 1) that can promote the formation of Norharman [51,52]. In addition, PhIP and 4,8-DiMeIQx in the SS3 samples were significantly higher than those of the other three samples (*p* < 0.05). This may be because some of the precursors in bacon, such as creatine (Figure 2), are released more easily from the muscle and participate in the formation of PhIP and 4,8-DiMeIQx, as the KCl replacement rate increases. Gibis et al. [44] also found that the PhIP content increased, and the creatine content declined in bacon after pan-frying.

## 4. Conclusions

This study showed that the HAA contents and quality characteristics of bacon are significantly affected by the partial replacements of NaCl by KCl. Bacon treated with SS3 caused a decrease in the Na^+^ content, moisture, a_w_, pH, L* value, and sensory saltiness and an increase in the K^+^ content, a* value, and sensory bitterness but did not affect the b* value, sensory redness, and sensory off-odor. The CS samples had the lowest TBARS and carbonyl contents. The different KCl-substituting rates had no significant effect on the creatine content in the marinating process but declined the creatine content after smoking and frying. An increase in the KCl-substituting rates led to an increase in the HAA contents in bacon. Moreover, the nonpolar HAA contents in bacon were higher than that of the polar HAA contents, and replacing NaCl with KCl mainly impacted the nonpolar HAA contents, especially Norharman. So, partially replacing NaCl with KCl accelerated the HAA formation in bacon. Therefore, in further studies, we will try to change the processing conditions and add some ingredients to reduce the HAA formation and lipid oxidation in low-Na^+^ content bacon.

## Figures and Tables

**Table 1:** Influence of the partial replacements of NaCl by KCl on the Na^+^ and K^+^ contents, moisture, water activity, pH, and color of bacon.

	CS	SS1	SS2	SS3
Potassium content (g/100 g)	0.08 ± 0.01 ^D^	0.17 ± 0.02 ^C^	0.29 ± 0.01 ^B^	0.40 ± 0.01 ^A^
Sodium content (g/100 g)	1.05 ± 0.01 ^A^	0.89 ± 0.03 ^B^	0.75 ± 0.01 ^C^	0.62 ± 0.03 ^D^
Moisture (g/100 g)	46.52 ± 2.65 ^A^	40.71 ± 1.20 ^B^	41.61 ± 1.32 ^B^	38.71 ± 1.46 ^B^
a_w_	0.971 ± 0.001 ^A^	0.965 ± 0.002 ^B^	0.965 ± 0.003 ^B^	0.967 ± 0.001 ^B^
pH	6.26 ± 0.02 ^A^	6.20 ± 0.01 ^B^	6.22 ± 0.02 ^B^	6.18 ± 0.01 ^C^
L*-value	56.24 ± 2.50 ^A^	51.81 ± 2.25 ^B^	51.00 ± 3.54 ^B^	51.71 ± 1.34 ^B^
a*-value	11.46 ± 1.31 ^B^	11.39 ± 1.14 ^B^	14.34 ± 1.14 ^A^	14.05 ± 0.73 ^A^
b*-value	9.67 ± 0.45 ^A^	11.01 ± 1.12 ^A^	11.08 ± 0.72 ^A^	10.70 ± 0.13 ^A^

^A–D^ Means within the same row with different superscripts showing significant differences (*p* < 0.05). CS, control (100% NaCl); SS1, 90% NaCl + 10% KCl; SS2, 80% NaCl + 20% KCl; and SS3, 70% NaCl + 30% KCl.

**Table 2:** Influence of the partial replacements of NaCl by KCl on the sensory qualities of bacon.

	CS	SS1	SS2	SS3
Saltiness	4.27 ± 0.32 ^A^	4.35 ± 0.36 ^A^	4.38 ± 0.33 ^A^	3.96 ± 0.41 ^B^
Redness	3.62 ± 0.25 ^A^	3.81 ± 0.29 ^A^	3.96 ± 0.28 ^A^	3.65 ± 0.36 ^A^
Bitterness	2.15 ± 0.35 ^B^	2.04 ± 0.39 ^B^	2.15 ± 0.30 ^B^	3.31 ± 0.41 ^A^
Off-odor	2.04 ± 0.23 ^A^	2.19 ± 0.35 ^A^	2.23 ± 0.41 ^A^	2.00 ± 0.41 ^A^

^A,B^ Means within the same row with different superscripts showing significant differences (*p* < 0.05). CS, control (100% NaCl); SS1, 90% NaCl + 10% KCl; SS2, 80% NaCl + 20% KCl; and SS3, 70% NaCl + 30% KCl.

**Table 3:** Influence of the partial replacements of NaCl by KCl on the HAAs content (ng/g) of pan-fried bacon.

	CS	SS1	SS2	SS3
Norharman	5.40 ± 0.57 ^C^	15.64 ± 1.21 ^B^	17.83 ± 1.34 ^A^	17.73 ± 0.84 ^A^
Harman	0.19 ± 0.02 ^C^	0.55 ± 0.04 ^B^	1.09 ± 0.09 ^A^	1.16 ± 0.06 ^A^
AαC	1.66 ± 0.25 ^A^	1.67 ± 0.16 ^A^	1.69 ± 0.13 ^A^	1.74 ± 0.08 ^A^
MeAαC	n.d.	n.d.	n.d.	n.d.
Nonpolar HAAs	7.25 ± 0.84 ^C^	17.86 ± 1.43 ^B^	20.62 ± 1.55 ^A^	20.64 ± 0.99 ^A^
IQ	0.17 ± 0.02 ^A^	0.18 ± 0.02 ^A^	0.17 ± 0.03 ^A^	0.18 ± 0.01 ^A^
IQx	0.39 ± 0.04 ^A^	0.38 ± 0.04 ^A^	0.38 ± 0.03 ^A^	0.40 ± 0.02 ^A^
7,8-DiMeIQx	0.76 ± 0.06 ^A^	0.76 ± 0.07 ^A^	0.77 ± 0.06 ^A^	0.80 ± 0.04 ^A^
4,8-DiMeIQx	0.51 ± 0.05 ^B^	0.57 ± 0.06 ^A,B^	0.53 ± 0.04 ^B^	0.62 ± 0.03 ^A^
MeIQ	n.d.	n.d.	n.d.	n.d.
MeIQx	0.81 ± 0.12 ^A^	0.77 ± 0.08 ^A^	0.80 ± 0.06 ^A^	0.83 ± 0.04 ^A^
PhIP	1.99 ± 0.15 ^B^	1.84 ± 0.18 ^B^	2.08 ± 0.10 ^A,B^	2.38 ± 0.11 ^A^
Phe-P-I	n.d.	n.d.	n.d.	n.d.
Polar HAAs	4.62 ± 0.55 ^B^	4.61 ± 0.46 ^B^	4.71 ± 0.27 ^B^	5.32 ± 0.25 ^A^
Total HAAs	11.87 ± 1.38 ^C^	22.47 ± 1.89 ^B^	25.32 ± 1.81 ^A,B^	25.96 ± 1.23 ^A^

n.d.: the levels were lower than the limit of detection. ^A–C^ Means within the same row with different superscripts showing significant differences (*p* < 0.05). CS, control (100% NaCl); SS1, 90% NaCl + 10% KCl; SS2, 80% NaCl and 20% KCl; and SS3, 70% NaCl + 30% KCl. PhIP: 2-amino-1-methyl-6-phenylimidazo[4,5-b]-pyridine; IQ: 2-amino-3-methyl-imidazo[4,5-f]-quinoline; MeIQ: 2-amino-3,4-dimethyl-imidazo[4,5-f]-quinoline; HAA: heterocyclic aromatic amine.

## Data Availability

The data presented in this study are available within the article.
